# Mechanisms of immune-related adverse events associated with immune checkpoint blockade: using germline genetics to develop a personalized approach

**DOI:** 10.1186/s13073-019-0652-8

**Published:** 2019-06-20

**Authors:** Zia Khan, Christian Hammer, Ellie Guardino, G. Scott Chandler, Matthew L. Albert

**Affiliations:** 1 Genentech Inc, 1 DNA Way, South San Francisco, CA 94080 USA; 2 Present address: Insitro, 279 East Grand Avenue, South San Francisco, CA 94080 USA

## Abstract

Personalized care of cancer patients undergoing treatment with immune checkpoint inhibitors will require approaches that can predict their susceptibility to immune-related adverse events. Understanding the role of germline genetic factors in determining individual responses to immunotherapy will deepen our understanding of immune toxicity and, importantly, it may lead to tools for identifying patients who are at risk.

## Immune checkpoint inhibitors and immune-related adverse events

Immune checkpoint inhibitors that block CTLA-4 (cytotoxic T-lymphocyte-associated protein 4), PD1 (programmed death 1), or PD-L1 (programmed death-ligand 1) have demonstrated significant promise in the clinic across a range of cancer indications [1]. In addition to their role in limiting immune responses against tumors, CTLA-4 and PD-1 are important immune checkpoints that contribute to the regulation of peripheral tolerance of tissue-specific self-antigens. Therapeutic blockade of these checkpoints results in a disruption of the balance between tolerance and immunity. In the clinic, this disruption manifests itself in the form of immune-related adverse events (irAEs), which are toxicities associated with checkpoint inhibitors that are autoimmune or autoinflammatory in origin. These toxicities differ in their severity, grade, and tolerability. Patients and their clinicians face challenging and important questions related to the use of checkpoint inhibitors. Does the benefit of therapy outweigh the risk of irAEs? If yes, how can a clinician proactively manage a patient who goes on to develop these toxicities? Should cancer patients with autoimmune disease be excluded from receiving this class of therapeutics? Personalized care requires urgent answers to these questions.

A growing body of literature is focused on characterizing irAEs and identifying new ways to manage patients who experience such events. Guidelines have emerged for grading and managing several broad classes of irAEs [2]. Notably, irAEs can affect virtually any tissue, with major targets including the skin, gastrointestinal tract, and endocrine organs. Moreover, differences in irAE occurrence exist across checkpoint inhibitors as a result of their differing mechanisms of action. Anti-CTLA-4 agents work by enhancing T-cell priming, whereas blockade of PD-1 or PD-L1 is thought to act by re-invigorating pre-existing CD8 T-cell responses [1]. In general, irAEs are more common with anti-CTLA-4 treatment than with anti-PD-1 or anti-PD-L1, probably reflecting their distinct roles in immune regulation [3]. Management guidelines for severe irAEs recommend treatment discontinuation or use of immunosuppressive therapies such as corticosteroids. It remains unclear whether these approaches limit the efficacy of immune checkpoint blockade and whether there is increased risk for new irAEs after restarting treatment [4]. Potentially life-threatening, high-grade irAEs such as myocarditis occur very rarely, but are of significant clinical concern. Strikingly, irAEs such as type 1 diabetes and inflammatory arthritis persist beyond the cessation of immune checkpoint blockade [5]. Some classes of irAEs may be associated with efficacy; for example, there is evidence that dermatological irAEs, such as vitiligo, might indicate general activation of the immune system [2]. Overall, observations relating to irAEs reveal a complex picture, which is why predicting risk for irAEs will require insight into their underlying mechanisms.

## What are the mechanisms through which irAEs might arise?

Much of what is known about the mechanisms that are thought to underlie irAEs is derived from pre-clinical and clinical studies of autoimmunity and autoinflammation [6]. An important observation made by these studies is that self-reactive T cells and B cells escape deletion by central tolerance [7]. Several molecular mechanisms limit the activity of these cells in the periphery, including the engagement of the immune checkpoints CTLA-4 and PD-1. Blockade of these checkpoints may enable T-cell activation following recognition of self-antigens, which can manifest as irAEs in the contexts and tissues in which these checkpoints normally function. Although patients with a medical history of autoimmune disease were excluded from clinical trials testing checkpoint inhibitors, sub-clinical autoimmunity might contribute to irAE occurrence. Notably, pre-existing autoantibodies have not been consistently detected in patients that develop irAEs [6], but no systematic evaluation has been performed. One proposed mechanism for irAE initiation involves a role for dysbiosis, in which exposure of microbiome-derived products can trigger an innate immune response, possibly leading to the activation of self-reactive immune cells. Intriguingly, features of the microbiome have been linked to CTLA-4-induced colitis in pre-clinical and clinical settings [8]. Epitope spreading may also contribute to irAEs as a result of the cross-reactivity of self- and tumor antigens, and is hypothesized to underlie checkpoint-inhibitor-induced myocarditis [6]. Although these mechanisms are important to autoimmunity, there are substantial challenges in distilling them into immune monitoring assays and predictive models.

## What can human genetics offer?

In addition to environmental factors, germline genetic factors contribute significantly to autoimmune disease risk [9]. Genome-wide association studies (GWAS) have identified genetic variants that confer risk or protection from autoimmune disease. Because the mechanisms underlying irAEs are thought to be driven by autoimmunity, these observations raise the question of whether germline genetic variation also impacts risk for irAEs. Although pre-clinical models have shown that blockade or genetic deletion of CTLA-4 or PD-(L)1 can increase the rate of autoimmunity in mice of vulnerable genetic backgrounds, this same observation has not yet been replicated in humans [6]. This link cannot be established easily because autoimmune diseases are highly polygenic and many variants across the genome contribute to genetic risk. One of the important features of the genetics of autoimmune disease is that variants in the major histocompatibility complex (MHC) locus are strongly associated with disease risk [9]. Most of these associations are mediated by human leukocyte antigen (HLA) genes, which play a central role in antigen presentation and immune tolerance. Variants outside the MHC locus are enriched in non-coding regions of the genome and most often display small effect sizes, making it difficult to interpret the effect of a single disease-associated variant. One way forward is to use the variants identified by autoimmune disease GWAS to generate individual-level polygenic risk scores [10]. If these scores are predictive of irAE occurrence, it could be inferred that shared genetic factors impact autoimmune disease and irAE risk. Polygenic risk scores may also capture the genetic component of an individual’s cancer-immune set point or immunological status, which may impact an individual’s response to immunotherapy [1].

Genome-wide single nucleotide polymorphism (SNP) data collected from patients treated with checkpoint inhibitors can also be used to identify variants in the genome that are associated with irAE risk or protection. We expect such efforts to be productive because of the strong influence of genetic variation on autoimmunity. This approach has two benefits. First, the genetic variants that are identified can be used to construct polygenic risk scores that can provide patients and clinicians with a personalized score that measures genetic risk for an irAE. Second, the variants and genomic loci found by this approach may highlight genes and immune pathways that modify irAE risk. Such genetic ‘hits’ can serve as the basis of studies looking to determine the mechanisms by which irAEs arise, and may also provide new insights into the mechanism of action for the desired on-target killing of tumor cells. For this approach to be successful, patient numbers will have to be sufficient to identify genetic factors that are associated with irAEs and to overcome heterogeneity in environmental exposures and treatment regimens. To this end, low-grade irAEs, which tend to be less important clinically, will be useful to increase statistical power, as they are more common and are possibly driven by the same autoimmune mechanisms as high-grade events. Ultimately, human genetic studies of irAEs will require the establishment of an international consortium and registry to coordinate data sharing and integration. Such efforts can be designed so that only summary level results leave an institution and no individual level data are shared, and due to the decreasing cost of genotyping arrays, such large-scale efforts are now feasible.

## Conclusions

As checkpoint inhibitors and immune therapies emerge as important treatments for cancer, personalized care will require approaches to predict the risk of irAEs. Human genetics provides powerful tools that can allow us to better understand the mechanisms of on-target tumor killing and off-target immune toxicities. Polygenic risk scores may provide important data that can be used by clinicians to optimize the benefit for each individual patient, and have the potential to contribute to predictive models of checkpoint-inhibitor treatments. Insights provided by human genetics into the immune mechanisms that are impacted by checkpoint inhibition may guide both the selection of targets for immunotherapy and the development of strategies to stratify patients.

## Abbreviations

CTLA-4: Cytotoxic T-lymphocyte-associated protein 4
GWAS: Genome-wide association study
irAE: Immune-related adverse event
MHC: Major histocompatibility complex
PD1: Programmed death 1
PD-L1: Programmed death-ligand 1
